# Inflammatory Myofibroblastic Tumor: A Rare Case Report

**DOI:** 10.7759/cureus.36579

**Published:** 2023-03-23

**Authors:** Nicholas D Luke, Samantha Gottlieb, Julia Brothers, Stephen Winikoff

**Affiliations:** 1 Medicine, St. George's University School of Medicine, True Blue, GRD; 2 Anesthesiology, St. Joseph's Regional Medical Center, Paterson, USA

**Keywords:** general anesthesia laryngoscopy, hemoptysis, pediatric anesthesiology, pediatric surgery, tracheal tumor

## Abstract

Inflammatory myofibroblastic tumors (IMTs) are rare benign tumors that can occur anywhere in the body, most commonly in the pediatric and young adult populations. The gold standard treatment is surgical resection, possibly along with chemotherapy and/or radiotherapy. IMTs have a high recurrence rate and may present with secondary symptoms, such as hemoptysis, fever, and stridor. We present a 13-year-old male patient with hemoptysis for one month who was subsequently diagnosed with an obstructing IMT of the trachea. The preoperative assessment showed the patient was not in acute distress and could protect his airway, even when lying flat. The treatment plan was discussed with the otolaryngologist, to keep the patient spontaneously breathing throughout the case. Anesthesia was induced with boluses of midazolam, remifentanil, propofol, and dexmedetomidine. Doses were adjusted as needed. Glycopyrrolate was also given to limit the patient's secretions before initiating the surgical procedure. The FiO_2_ was kept under 30% as tolerated to reduce the risk of airway fire. During surgical resection, the patient was kept spontaneously breathing, and paralytics were avoided. Due to high tumor vascularity and inability to obtain hemostasis, the patient was kept intubated and on ventilation post-operatively until definitive treatment could be performed. On postoperative day 3, the patient returned to the operating room due to a worsening condition. He was found to have a partial obstruction of the right mainstem bronchus by the tumor. More of the tumor was debulked, and he remained intubated above the level of the debulked mass. The patient was then transferred to a higher acuity institution for advanced care. After the transfer, the patient underwent a carinal resection on cardiopulmonary bypass. This case provides insight into successfully sharing the airway during tracheal tumor resection, emphasizing minimizing the risk of airway fire and constant communication with the surgeon.

## Introduction

A subtype of a tracheal tumor is an inflammatory myofibroblastic tumor (IMT). This rare and benign tumor may occur anywhere in the body and is comprised of cells from the immune system, smooth muscle, and connective tissue. It is predominantly found in the pediatric and young adult population and most commonly occurs in the lungs, abdomen, pelvis, and retroperitoneum [1]. While IMTs are usually painless, they may cause symptoms due to local mass effects [2]. If left alone, these tumors can progressively grow and cause obstruction of the airway, which in turn can be a medical emergency. The use of imaging can depict infiltrative growth; however, it is nonspecific until histopathological proven [2]. These tumors further complicate patient prognosis due to high reoccurrence rates and the likelihood of metastasis to distant sites within the body [3]. The primary treatment of IMTs involves surgical excision. However, if the patient cannot tolerate surgery, radiation, laser ablation, or cryotherapy can be utilized as an alternative. If anaplastic lymphoma kinase (ALK) gene arrangements are identified, using the ALK inhibitor Crizotinib is also a viable option [3].

## Case presentation

**Figure 1:**
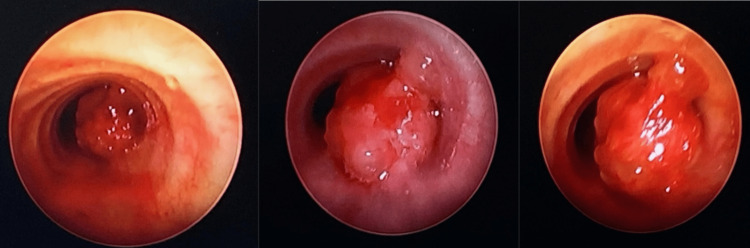
The bronchoscopic view of the tracheal tumor. The large size of the tumor caused the patient to be stimulated intermittently upon airway manipulation during the procedure.

**Figure 2:**
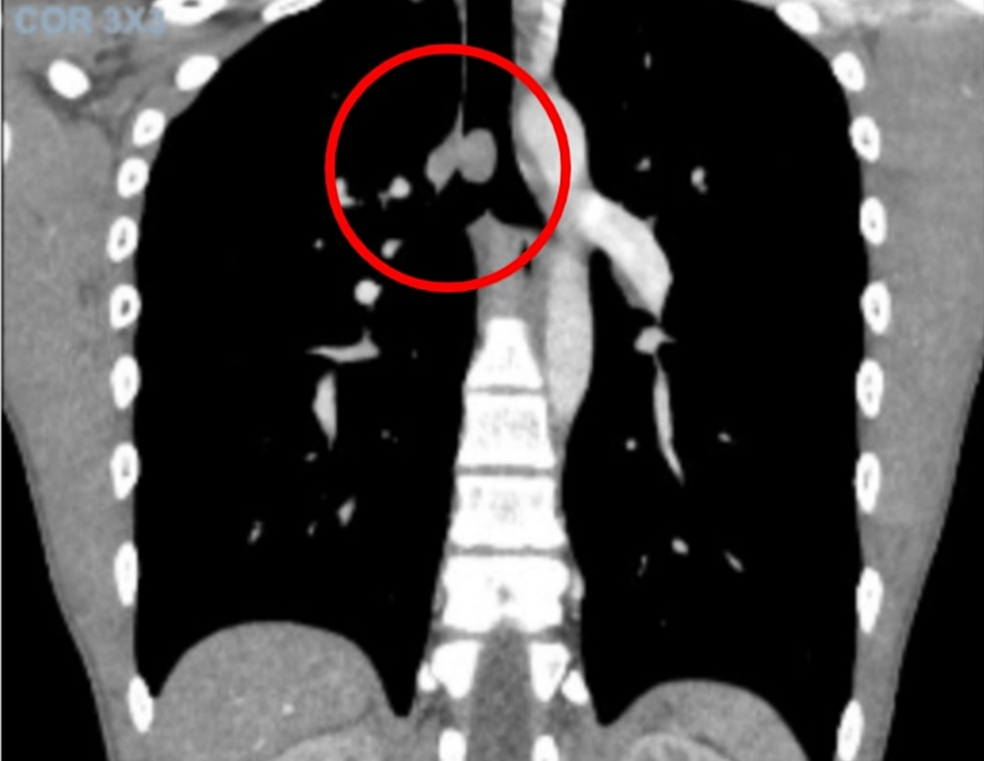
A computed tomography image with contrast depicting an endobronchial mass seen within the distal trachea (red circle) without atelectasis or infiltration. The mass involving the proximal part of the right bronchus was moderately obliterating the tracheal lumen and measured approximately 8 x 11 x 19 millimeters.

**Figure 3:**
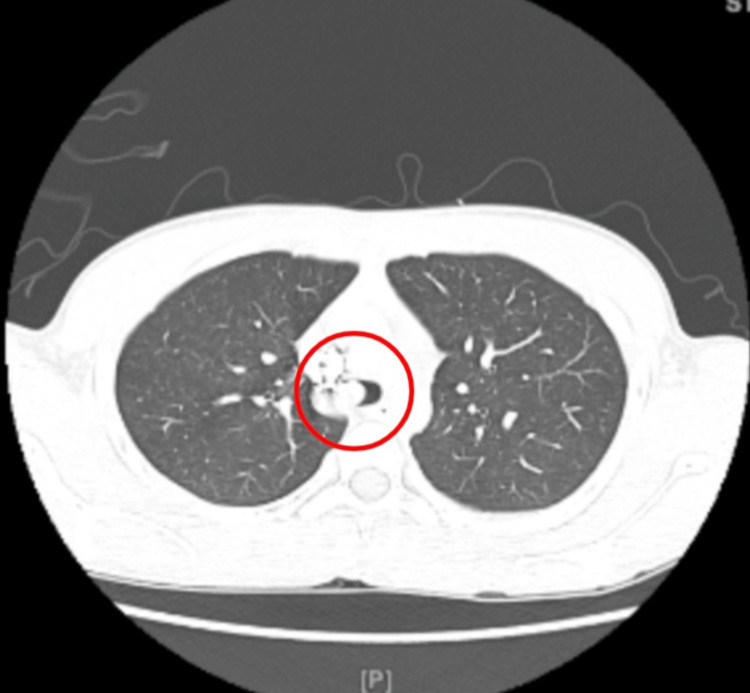
A computed tomography angiographic image with intravenous contrast depicting a soft tissue mass in the trachea measuring 19 millimeters (red circle). It did not demonstrate significant arterial enhancement.

We present a 13-year-old male with hemoptysis and stridor for one month. Before the initial surgical intervention, bronchoscopy and radiographic imaging demonstrated a lower airway mass (Figures 1-3). The patient did not have difficulty in breathing when lying flat and was not in respiratory distress during this hospital admission. The patient's American Society of Anesthesiologists (ASA) score was one. The pulmonary function testing showed a Forced Expiratory Volume in the First Second/Forced Vital Capacity (FEV1/FVC) ratio of 23%, and the FVC was 0.84 liters. All vital signs were within normal limits, and the lungs were clear to auscultation bilaterally.

 

The patient was taken to the operating room for a rigid bronchoscopy, biopsy, and debulking of the mass. Before surgery, the patient was not in respiratory distress, demonstrated no difficulty breathing or protecting the airway, and could lie flat. Once in the operating room, standard ASA monitors were applied, along with end-tidal CO_2_ monitoring connected to a nasal cannula, and then eventually switched to the ENT laryngoscope. The plan was discussed with the ENT surgeon to keep the patient spontaneously breathing throughout the case. Anesthesia was induced with boluses of midazolam (2 mg), remifentanil, propofol, and dexmedetomidine were initiated, and doses were adjusted as needed. Glycopyrrolate (0.2 mg) was also given to limit patient secretions at the start of the case. FiO_2_ was kept under 30% as tolerated to reduce the risk of airway fire, and water was immediately available on the OR table in case of emergency. During the surgical procedure, the patient required intermittent intubation by the surgeon with a 6.0 micro-laryngoscopy endotracheal tube (MLT) to facilitate the view of the airway.

The pathology report demonstrated a cellular spindle tumor with a prominent storiform growth pattern. No necrosis or cellular atypia was noted. A variable number of small lymphocytes and a few plasma cells are scattered throughout the biopsy specimen. The focal overlying squamous epithelium was also present, and the submucosal area showed an increased number of inflammatory cells, including small lymphocytes, neutrophils, mast cells, and rare eosinophils. On immunostaining, the spindle cells are positive for muscle-specific actin, and many spindle cells were also positive for ALK.

Upon case completion, there was a concern for bleeding from the tumor site despite multiple attempts to attain hemostasis with cautery, epinephrine, and laser. The anesthesiologist and surgeon decided to intubate the patient for post-operative management. The patient was then intubated by a surgeon with 5.0 cuffed MLT that was advanced by the surgeon into the L mainstem bronchus, past the bleeding site in the right mainstem bronchus. The patient was brought to PICU in stable condition.

Post-operative day 3, the patient returned to the operating room due to worsening condition. The patient was found to have a partial obstruction of the right mainstem bronchus by the tumor. Once discovered, the plan was to transfer the patient to higher support for advanced care. Before the transfer, more of the tumor was debulked, and the patient remained intubated above the level of the debulked mass. Tracheostomy was not performed because the patient was stabilized after the debulking procedure, and there was no intention of keeping the patient on the ventilator for an extended period of time. After the transfer; the patient underwent a carinal resection on cardiopulmonary bypass.

## Discussion

IMTs have an incidence of 0.04% and are equal in males and females within the lungs [4]. Diagnosis may be missed due to rarity, so clinicians must rule them out in patients with stridor and hemoptysis. Clinicians should also know these tumors are locally invasive and may reoccur and metastasize. Despite this, the prognosis is good, with a five-year survival rate of 74% to 91% [4].

The standard approach to treat IMTs is surgical resection. For locally aggressive and invasive tumors with positive margins and surgically unresectable tumors, adjuvant chemotherapy and/or radiation therapy may be considered. Na and Park utilized glucocorticoids and radiotherapy in their patient, which resulted in a ~25% decrease in the tumor size, resolution of hematuria (from metastasis to the kidney), and overall improvement of symptoms in the patient at one-month follow-up [5]. In another study, Tao et al. utilized non-steroidal anti-inflammatory drugs, methotrexate, and cisplatinum, which resulted in a non-palpable tumor that could not be identified on imaging [6]. In our patient, the decision was made to perform carinal surgery on a cardiopulmonary bypass due to the size and location of the mass. Hoseok et al., for instance, used carinal resection on a four-year-old patient with success; since the tumor may reoccur and difficult to resect cases, the carinal procedure may be a better option. They also emphasized that IMTs that extend beyond a single organ have a much higher chance of recurrence, despite having adequate margins during the resection [7]. If a carinal surgery is impossible, more frequent postoperative observation may be needed along with adjuvant chemotherapy.

Another essential feature of this case is the elevated risk of airway fire. Although rare, they are extremely dangerous when the surgery is performed within the airway. Fire needs three components: oxygen, ignition source, and fuel. Using cautery or laser with FiO_2_ >30% in the airway provides the perfect environment for a fire to ignite. Anesthetic gases may also impact the ignition of airway fire, even if no leak is present [8]. In cases that involve airway IMT resections, special care should be taken to minimize the risk of airway fire, including constant communication with the surgeon.

## Conclusions

IMTs are extremely rare and may have variable histologic and clinical presentations. These tumors are benign but can cause secondary symptoms due to mass effects, such as obstructed airways. The standard of treatment is surgical resection; however, chemotherapy, radiotherapy, and ALK inhibitors have also been utilized with good efficacy. For best outcomes, it is important to have meticulous and early planning and good communication with the surgeon for all aspects of perioperative care. In our presenting patient, the tumor was eventually removed with a carinal resection after several debulking and resection procedures. Intermittent intubation with the micro-laryngoscopy endotracheal tube placement was needed to visualize the airway and allow for the anesthesiologist and surgeon to work in unison to provide excellent patient care and safety.
